# Prevalence of antepartum hemorrhage in women with placenta previa: a systematic review and meta-analysis

**DOI:** 10.1038/srep40320

**Published:** 2017-01-09

**Authors:** Dazhi Fan, Song Wu, Li Liu, Qing Xia, Wen Wang, Xiaoling Guo, Zhengping Liu

**Affiliations:** 1 Department of Obsterics, South Medical University Affiliated Maternal & Child Health Hospital of Foshan, Foshan, Guangdong, 528000, China; 2 Department of Epidemiology and Biostatistics, School of Public Health, Anhui Medical University, Hefei, Anhui, 230032, China; 3 School of Integrated Traditional and Western Medicine, Anhui University of Chinese Medicine, Hefei, Anhui, 230038, China; 4 Department of Library, the First Affiliated Hospital, College of Medicine, Zhejiang University, Hangzhou, Zhejiang, 310003, China

## Abstract

Antepartum hemorrhage (APH) is an important cause of perinatal mortality and maternal morbidity in pregnant women with placenta previa in the world. However, the epidemiological characteristics are not completely understood. We performed an initial systematic review and meta-analysis to assess the prevalence of APH in pregnant women with placenta previa. It was totally performed following the Preferred Reporting Items for Systematic reviews and Meta-Analysis statement. PubMed, Elsevier Science Direct, and the Cochrane Library were searched before April 2016. A meta-analysis with a random-effects model based on a proportions approach was performed to determine the prevalence. Stratified analyses, meta-regression method, and sensitivity analysis were utilized to analyze the heterogeneity. A total of 29 articles were included. The pooled overall prevalence of APH among pregnant women with placenta previa was 51.6% (95% CI 42.7–60.6) in a heterogeneous set of studies (*I*^2^ = 97.9). Correlation analysis found that there was a positive correlation between prevalence and percentage of multiparous (r = 0.534, P = 0.027) and a negative correlation between prevalence and survey year (r = −0.400, P = 0.031). In conclusion, the prevalence of APH was a high condition among pregnant women with placenta previa.

Antepartum hemorrhage (APH) defined as bleeding from the genital tract in the second half of pregnancy, is still an important cause of perinatal mortality and maternal morbidity in the world^1^. It is found that there is a strong relationship between APH and later development of intrapartum bleeding necessitating cesarean delivery^2^. Some women necessitate preterm cesarean section and hysterectomy for life-threatening APH, whereas others undergo elective cesarean section at term without hemorrhagic complications. In addition to unknown origin, the major causes of APH are placenta previa and abruption placenta^3^. Placenta previa is one of the most serious complications during pregnancy and is associated with numerous adverse maternal and fetal-neonatal complications. Many of these are direct consequences of maternal antepartum and intrapartum hemorrhage^4^. It was reported that the prevalence of APH in pregnant women with placenta previa are at an approximately ten times greater than that non-placenta previa women^5^. Importantly, the prevalence of placenta previa has been rising in parallel with the increasing rate of cesarean delivery and varies throughout the world and it has become a serious public health problem worldwide^6^,^7^.

Although APH is common in pregnant women with placenta previa, it has not been extensively studied. Studies have suggested that pregnant women with placenta previa experience higher rates of APH than the general women^2^,^8^,^9^. However, estimates of the prevalence vary across studies, from 20% to 90%^10^,^11^. The prevalence may vary due to the maternal age, previa location (anterior vs. posterior), previa types (complete vs. incomplete), the nature of the population studied, lifestyle habits, the use of different diagnosis criteria, and other potential characteristics^12^,^13^,^14^,^15^.

A reliable estimate of APH in placenta previa is important for informing efforts to prevent, treat, and identify causes of APH among pregnant women with placenta previa and would contribute to the planning and implantation of relevant public health strategies. Against this background, we conducted a systematic review and meta-analysis of published studies of APH to estimate the prevalence of APH among pregnant women with placenta previa.

## Results

### Search Results and Characteristics

**Figure 1:**
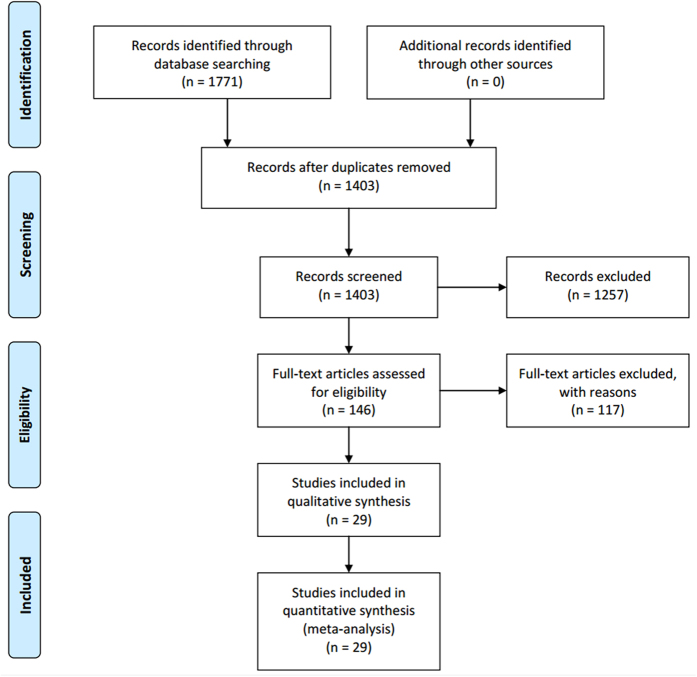
PRISMA flowchart showing the study selection process.

The detailed steps of the systematic review and meta-analysis process were given as a PRISMA and the identifying eligible studies were summarized in Fig. 1. Twenty-five retrospective and four prospective cohort studies involving a total of 4687 individuals were included in the study (Table 1). Fourteen^15^,^16^,^17^,^18^,^19^,^20^,^21^,^22^,^23^,^24^,^25^,^26^,^27^,^28^ took place in Asia, eight^5^,^29^,^30^,^31^,^32^,^33^,^34^,^35^ in North America, six^10^,^11^,^36^,^37^,^38^,^39^ in Europe, and one^40^ in Africa. The articles were published between 1985 and 2016, and the median number of participants per study was 162 (range, 40–703). When evaluated by STROBE quality assessment criteria, three studies received 9 points, eight received 8 points, eleven received 7 points, and seven received 6 points. The quality scores show that in general, and the studies considered to be of acceptable quality.

### Overall prevalence

**Figure 2:**
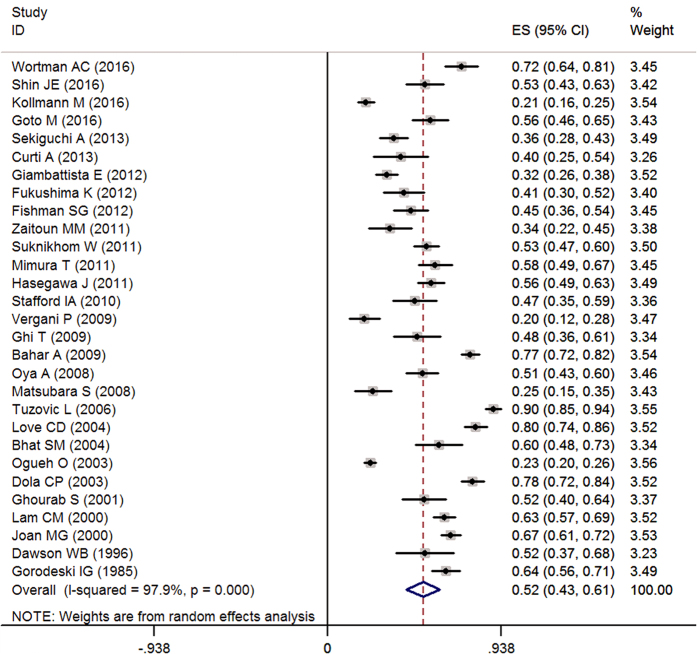
Forest plot of pooled estimated prevalence of APH with 95% CI.

Meta-analytic pooling of the prevalence estimates of APH reported by the 29 studies yielded a summary prevalence of 51.6% (2347/4687 individuals, 95% CI, 42.7–60.6%), with significant evidence of between study heterogeneity (Q = 1345.19, *P* < 0.001, *I*^*2*^ = 97.9%) (Fig. 2).

**Figure 3:**
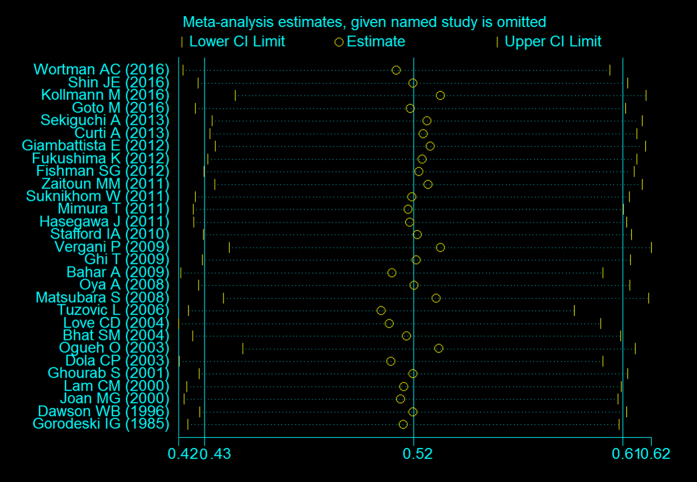
Sensitivity analysis for individual studies on the summary effect.

**Figure 4:**
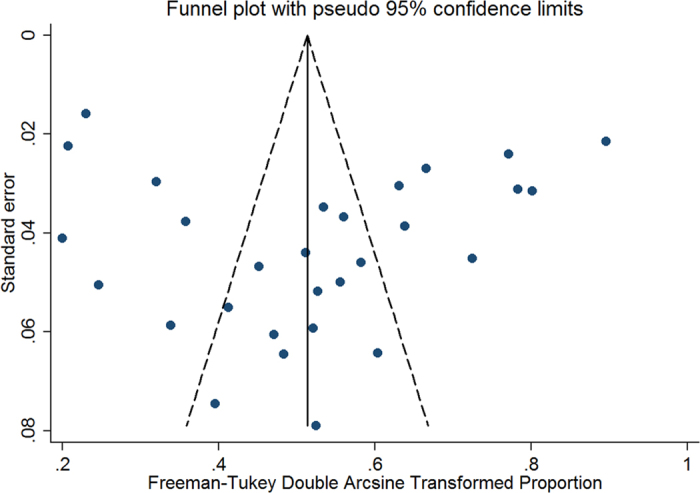
Funnel plot of the 29 studies included in the meta-analysis.

To confirm the stability and liability of the meta-analysis, sensitivity analysis was performed by calculating pooled APH prevalence again when any single study was deleted. Figure 3 showed that the corresponding pooled prevalence varied from 50.2% (42.0–58.5%) (when excluding Tuzovic L. *et al*.^11^) to 52.8% (44.0–61.6%) (when excluding Kollmann M. *et al*.^36^) and was not substantially altered. The statistically similar results reported that each single study didn’t influence the stability of overall APH prevalence estimate in this meta-analysis. Although visual inspection of the funnel plot revealed slightly asymmetry (Fig. 4), both Begg’s test (*z* = 1.33, *P* = 0.183) and Egg’s test (*t* = 0.41, *P* = 0.688) showed no potential risk of publication bias.

### Subgroup results

To provide a range of the APH prevalence estimates identified by the study design, estimates were stratified by retrospective and prospective cohort study. Summary prevalence estimates ranged from 52.8% for the retrospective study (2158/4129 individuals, 95% CI, 43.3–62.4%, *I*^*2*^ = 97.9%) to 43.8% for the prospective cohort study (189/558 individuals, 95% CI, 23.0–64.7%, *I*^*2*^ = 95.4%). Although studies by retrospective yielded higher than prospective (52.8% vs. 43.8%), there were no significant differences between estimates made using the retrospective and prospective cohort study (95% CI, 43.3–62.4% vs. 95% CI 23.0–64.7%), suggesting that variation between study design did not explain the heterogeneity in the observed APH prevalence estimates.

When evaluated by geographic region, the mean prevalence was high in Asia (53.4%, 1124/1981 individuals, 95% CI, 45.8–61.0%, *I*^*2*^ = 92.0%), intermediate in North America (53.2%, 696/1548 individuals, 95% CI, 34.1–72.2%, *I*^*2*^ = 98.3%) and Europe (48.5%, 505/1093 individuals, 95% CI, 20.3–76.7%, *I*^*2*^ = 99.3%), and low in Africa (33.8%, 22/65 individuals, 95% CI, 22.3–45.3%). In contrast, there were significant differences between Asia and Africa (95% CI, 45.8–61.0% vs. 95% CI 22.3–45.3%).

### Meta-regression

A high level of heterogeneity between studies and subgroups was observed. Meta-regression was carried out to explore potential sources of heterogeneity. Maternal age, gestational week, year of data collection, quality score, and percentage of anterior placenta, previous cesarean section, and multiparous, which may be potential sources of heterogeneity, were all tested by meta-regression method.

**Figure 5:**
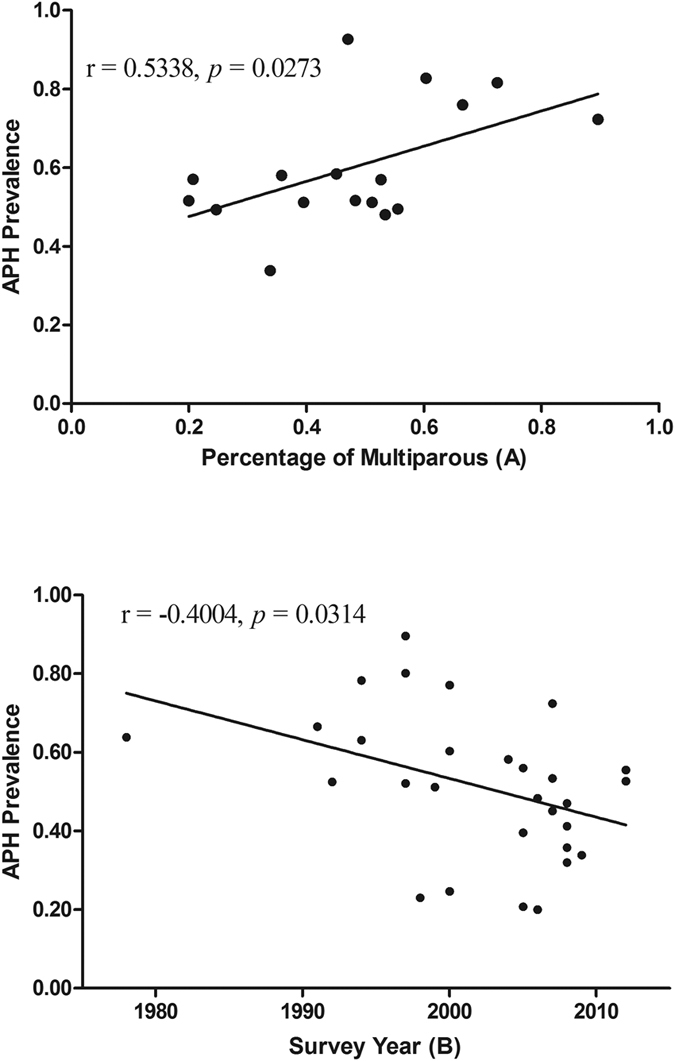
The correlation between APH prevalence and percentage of multiparous (**A**) and survey year (**B**).

The results of meta-regression were given in Table 2. Through the regression model, except for survey year (*P* = 0.033) and percentage of multiparous (*P* = 0.029), none of aforementioned variables was significantly associated with the detected heterogeneity. We, therefore, further tested the correlation between APH prevalence and potential sources. It was found that there was a positive correlation between APH prevalence and percentage of multiparous (r = 0.534, *P* = 0.027) and a negative correlation between APH prevalence and survey year (r = −0.400, *P* = 0.031) (Table 3 and Fig. 5).

## Discussion

This systematic review and meta-analysis of 29 studies involving 4687 pregnant women with placenta previa demonstrated that between 20% and 78% of women screened positive for antepartum hemorrhage (APH). The pooled estimated of the prevalence showed that more than half (51.6%) of pregnant women experience APH. Finding from this study demonstrated that APH was an important problem for pregnant women with placenta previa, and showed wide variation in the prevalence between studies.

To our knowledge, this review was the first time to conduct a meta-analysis reporting APH prevalence in pregnant women with placenta previa. Characteristics of studies that might affect these estimates were further investigated in this study. The pooled risk estimates indicated that survey year and multiparous were significantly associated with the APH prevalence for pregnant women with placenta previa. Given the high heterogeneity, it was best to consider the confidence interval rather than the pooled result. Because the development of APH has been involved in a higher risk of future delivery and greater long-term morbidity, these findings may affect the long-term health of obstetricians.

APH is a major cause of maternal and fetal morbidity and mortality complicating 2–5% of all pregnancies^2^. It is linked to an increased risk of emergency cesarean section, need for blood transfusion, maternal intensive care unit admission, hysterectomy, septicemia, thrombophlebitis, and even maternal death^29^,^41^. It may occur from the placental site, lesions of the cervix or vagina and occasionally fetal in origin^1^,^2^. The major causes of APH are placenta previa and abruption placenta. Other risk factors include marginal sinus bleeding, vasa previa, cord velamentous insertion, battledore placenta, cervicitis, genital trauma, tumours, infections, and coagulation defects^20^,^30^,^42^.

As the chief cause of APH in late pregnancy, the overall prevalence of placenta previa has been recently estimated to be approximately 5 per 1000 pregnancies by world region^7^. Women with placenta previa are at an approximately 4-fold increased risk of second trimester vaginal bleeding^42^ and some women necessitate preterm cesarean section and hysterectomy for life-threatening hemorrhage. The prevalence of APH reported in studies was considerably different among different countries in pregnant women with placenta previa. It was reported that the prevalence was 89.6% in Croatia^11^, 66.6% in Canada^5^, whereas the data was only 33.8% in Egypt^40^. In prospective studies, the prevalence of APH was 20.7%, 48.3%, 52.1%, and 55.6% in Austria^36^, Italy^38^, Saudi Arabia^25^, and Japan^17^, respectively. When assessed by geographic region, the prevalence was high in Asia (53.4%), intermediate in North America (53.2%) and Europe (48.5%), and low in Africa (33.8%) in this meta-analysis. In contrast, there were significant differences between Asia and Africa. However, the unbalanced distribution of studies (Asia: 14 and Africa: 1) would be undermined the statistical analyses, so it should be cautious and best to consider the confidence interval rather than the pooled result.

Differences among prevalence estimates in different countries in women with placenta previa may be due to a number of factors. For instance, demographic and socioeconomic characteristics^17^,^29^,^36^, cultural terms^22^, lifestyles^26^ and health statuses^27^ vary greatly between countries at different stages of development. Evidence has existed that normal gestational length was longer in white European than Black and Asian in nulliparous women with singleton live fetuses at the time of spontaneous labour^43^. With the increase of gestational age, the risk of APH will increase in pregnant women with placenta previa. It has been observed that the mean gestational age was only 34.2 weeks in placenta previa women in Egypt^40^. In addition, survey year^5^, sampling source and methods^10^,^38^, and sample size^22^,^35^ have a profound influence on the prevalence of APH.

Short cervical length, sponge-like echo in the cervix, placenta lacunae, and the lack of a clear zone are currently regarded as risk factors for APH. The APH incidence appears to have increased in relationship to the increasing rate of endometrial damage in pregnant women. Risk factors for endometrial damage include increasing maternal parity, induced labor, artificial abortion and the number of previous cesarean deliveries. This may explain why the positive correlation was found between APH prevalence and percentage of multiparous in placenta previa in our study. However, similarly result was not found in percentage of previous cesarean section. This discrepancy could reflect a lack of statistical power in this system review owing to the small number of studies.

The purpose of this study was to explore the overall prevalence of APH in pregnant women with placenta previa from 1985 through 2016. Interestingly, it was found that a negative correlation between APH prevalence and survey year. It does mean that as time goes on the mean prevalence will be becoming progressively decreased through epidemiological study. Although this review and meta-analysis have not been considered, we speculate that the main reasons for the decreased rate of APH may be the national health care systems and the improvement in diagnostic techniques and obstetric practice. Compared with fragile health care systems, strong health care systems can provide safe, timely, and appropriate cesarean delivery which is required to ensure optimal maternal and neonatal health outcomes in comprehensive emergency obstetric^44^. Since 2011, it was recommended that women with placenta previa with previous bleeding events should be admitted at or after 34 weeks’ gestation by the Royal College of Obstetricians and Gynaecologists^12^. The recommendation will decrease the risk of gestational complications (including APH) in pregnant women with placenta previa. However, the exact reasons that may explain this trend need confirmed in future studies.

The systematic review and meta-analysis included 29 studies with a 4687 individuals, and it showed no potential risk of publication bias. The overall quality of the studies included was all acceptable. And, the result of sensitivity analysis was not substantially altered. Nevertheless, limitations should be taken into account when interpreting the findings of this study. Firstly, just 29 articles were included in this meta-analysis, and only one study from Africa. The variability of sample size and unbalanced distribution of studies would be undermined the statistical analyses and estimation of the prevalence at global scale, so it should be cautious and best to consider the confidence interval rather than the pooled result. Secondly, most of publications (25/29) included in this study were retrospective observational studies, which were considered moderate evidence, and so the conclusions drawn in this analysis were restricted by this study type. Thirdly, significant heterogeneity was observed in the study, which was not surprising as heterogeneity often exists in such meta-analysis of overall prevalence^45^,^46^,^47^. Although subgroup and meta-regression analyses did indicate geographic region, survey year, and percentage of multiparous to explain the observed heterogeneity, the remainder among the studies could be unexplained by the variables examined. We propose that other factors, such as lifestyle habits, alcohol or coffee consumption, mental and physical inactivity may influence APH heterogeneity. Further analyses could not be performed, because of the limited information on these aspects. Finally, the results relied on aggregated published data. Further large-scale, multicenter prospective study using a single validated measured of APH in a random subset of participants would provide a more accurate estimate of the prevalence of APH in women with placenta previa.

In summary, in this systematic review, the summary estimate of the prevalence of APH among women with placenta previa was 51.9%, ranging from 20% to 78% depending on the studies. Further research is needed to identify effective strategies for preventing and treating APH among women with placenta previa.

## Methods

This study used standard methods for systematic reviews and meta-analysis of observational studies in accordance with the Preferred Reporting Items for Systematic Reviews and Meta-Analysis (PRISMA) and the Meta-analysis of observational studies in epidemiology (MOOSE) statements. The systematic review protocol has been published in the PROSPERO International Prospective Register of systematic reviews (http://www.crd.york.ac.uk/PROSPERO/), and the registration number is CRD42016037823^48^.

### Search strategy and selection criteria

The following online computer databases were searched: MEDLINE, Elsevier Science Direct, and the Cochrane Library using the subject terms: “placenta previa”, “Low-lying placenta”, “PP”, “hemorrhage”, “haemorrhage”, “vaginal bleeding”, “antepartum”. Relevant literature was also hand searched and identified based on the reference lists of original and review articles already deemed eligible for inclusion. The search language was limited to English, and the search was updated in April 2016. APH was defined as bleeding occurring after 20 weeks of gestation until delivery. The studies were included in our analysis if they meet the following criteria: studies either provided the number of cases of APH and sample size of pregnant women with placenta previa or sufficient information of crude prevalence of APH; and articles were published in English. The studies were excluded based on the following criteria: case series, letters, reviews and editorials; or the full data was not accessible even after request from the corresponding author. If multiple publications covered the same population, the most detailed one reporting the largest sample size was considered.

### Data extraction

After initial evaluation, two trained investigators (DF and SW) independently and carefully evaluated the articles and performed the data extraction according to a standardized form. The following variables were extracted: first author, publication year, study design (as defined by the original article), study period, geographic region, number or percentage of potential characteristics (previous cesarean sections, multiparous, anterior position of placenta), maternal age and gestational week at delivery (mean ± standard deviation or median, range), the number of APH and the total sample size. When discrepancies existed, discussion was performed or via consultation with another reviewer (QX) until a consensus was reached. When there were several involving the same population, only the most comprehensive publication was included. If necessary, the corresponding author of the published studies was contacted to provide relevant information for our analysis.

### Methodological quality assessment

The Reporting of Observational Studies in Epidemiology (STROBE) guideline was used to assess the methodological quality of each study included in systematic reviews and meta-analysis by two independently investigators (DF and QX)^49^. The items included five core components including sample population, sample size, participation rate, outcome assessment, and analytical methods to control for bias. Briefly, the item is assessed by scoring (low risk = 2, moderate risk = 1, high risk and unclear = 0) each bias type for each publication and the total score is used as the summary assessment of risk of bias. Detailed STROBE quality assessment. The total score, ranged from 0 to 10, represented the summary assessment of bias risk for each study and was used to assess the risk of bias for the individual studies^46^. When there was a disagreement, it was solved by consensus of the whole team.

### Statistical Analysis

Individual and pooled incidence and 95% confidence interval (95% CI) were calculated for each of all the included studies using the STATA 12.0 (Stata-Corp, College Station, TX, USA). Before performed an inverse-variance weighted, prevalence was transformed via the Freeman-Tukey double arcsine method^50^. The pooling prevalence estimates using random-effects meta-analysis that accounted for between-study heterogeneity. Statistical heterogeneity was assessed by the chi-square test on Q statistic, which was quantified by the I-square values, assuming that I-square values 25, 50 and 75% were nominally assigned as low, moderate, and high estimates, respectively^51^. To investigate potential sources of heterogeneity, stratified analyses and meta-regression were performed to find any possible sources using the following grouping variables: study design (retrospective vs. prospective), geographic region, study period, maternal age, gestational week, percentage of potential characteristics (previous cesarean sections, multiparous, and anterior position of placenta) and study quality. Sensitivity analysis was also performed by excluding each study at a time to assess whether one or more studies influenced the overall results. Publication bias was assessed first by visually inspecting the distribution of observed studies on a funnel plot^52^. Then, Begg’s rank correlation test^53^ and Egger’s linear regression^54^ were utilized to quantify the degree of bias illustrated in the funnel plot. And P ≤ 0.05 was indicated the presence of statistically significant.

## Additional Information

**How to cite this article**: Fan, D. *et al*. Prevalence of antepartum hemorrhage in women with placenta previa: a systematic review and meta-analysis. *Sci. Rep.* **7**, 40320; doi: 10.1038/srep40320 (2017).

**Publisher's note:** Springer Nature remains neutral with regard to jurisdictional claims in published maps and institutional affiliations.

## Figures and Tables

**Table 1:** Selected characteristics of the studies included in this systematic review and meta-analysis.

Source	Country	Survey Period	Cases	Total	Prevalence (%)[95% CI]	Age (year)*	GA (week)*	AP	PCS	MP	Study	QS
Wortman AC^29^	USA	2002–2012	71	98	72.4 (63.6,81.3)	30.8 ± 6.1	38.1 ± 2.3	18	21	80	Rep	8
Shin JE^16^	Korea	2010–2014	49	93	52.7 (42.5,62.8)	35.5 ± 4.2	37.0 ± 2.4	14	23	53	Rep	7
Kollmann M^36^	Austria	1993–2012	68	328	20.7 (16.3,25.1)	31.6 (17–46)	35.6 (23–41)	—	—	187	Pro	8
Goto M^17^	Japan	2009–2014	55	99	55.6 (45.8,65.3)	35.5 (25–49)	—	15	15	49	Pro	8
Sekiguchi A^28^	Japan	2004–2012	58	162	35.8 (28.4,43.2)	33.5 ± 4.4	37.4 (17–38)	31	19	94	Rep	9
Curti A^30^	USA	2001–2010	17	43	39.5 (24.9,54.1)	33.8 (25–44)	37.1 (28–40)	—	11	22	Rep	7
Giambattista E^37^	Italy	2006–2010	79	247	32.0 (26.2,37.8)	35.5 ± 4.8	—	—	51	—	Rep	6
Fukushima K^18^	Japan	2006–2010	33	80	41.2 (30.5,52.0)	33.5 (23–43)	37.0 (28–38)	5	12	—	Rep	6
Fishman SG^31^	USA	2002–2010	51	113	45.1 (36.0,54.3)	—	36.0 (34–37)	33	19	66	Rep	6
Zaitoun MM^40^	Egypt	2008–2010	22	65	33.8 (22.3,45.3)	31.3 ± 4.4	34.2 ± 2.4	—	21	22	Rep	6
Suknikhom W^19^	Thailand	2006–2008	110	206	53.4 (46.6,60.2)	33.4 ± 4.6	—	71	39	99	Rep	8
Mimura T^20^	Japan	2000–2008	67	115	58.3 (49.2,67.3)	33.4 ± 4.3	35.2 ± 2.4	9	12	—	Rep	7
Hasegawa J^21^	Japan	2000–2009	102	182	56.0 (48.8,63.3)	33.6 (23–43)	36 (22–37)	24	—	—	Rep	7
Stafford IA^32^	USA	2007–2009	32	68	47.1 (35.2,58.9)	31.9 ± 5.8	—	32	35	63	Rep	8
Vergani P^10^	Italy	2003–2008	19	95	20.0 (12.0,28.0)	34.2 ± 4.1	36.8 ± 3.6	46	—	49	Rep	9
Ghi T^38^	Italy	2005–2007	29	60	48.3 (35.7,61.0)	34.9 ± 4.5	34.7 ± 2.3	—	49	31	Pro	8
Bahar A^22^	Saudi Arabia	1996–2005	236	306	77.1 (72.4,81.8)	30.6 (16–49)	—	—	165	—	Rep	6
Oya A^23^	Japan	1993–2007	66	129	51.2 (42.5,59.8)	32.1 ± 5.0	35.9 ± 2.9	—	16	66	Rep	7
Matsubara S^24^	Japan	1994–2005	18	73	24.7 (14.8,34.5)	31.4 (28–34)	37 (36–39)	—	9	36	Rep	7
Tuzovic L^11^	Croatia	1992–2001	181	202	89.6 (85.4,93.8)	—	—	65	20	146	Rep	8
Love CD^39^	UK	1994–2000	129	161	80.1 (74.0,86.3)	—	36.4 ± 5.9	—	—	—	Rep	7
Bhat SM^15^	Oman	1998–2002	35	58	60.3 (47.8,72.9)	30.6 ± 5.9	35.0 ± 3.4	—	8	48	Rep	7
Ogueh O^33^	Canada	1997–1999	162	703	23.0 (19.9,26.2)	31.3 ± 4.8	39.4 ± 1.8	—	81	—	Rep	6
Dola CP^34^	USA	1990–1997	137	175	78.3 (72.2,84.4)	30.8 ± 5.7	35.3 ± 3.5	62	68	—	Rep	9
Ghourab S^25^	Saudi Arabia	1995–2000	37	71	52.1 (40.5,63.7)	29.5 ± 3.8	35.2 ± 4.3	40	—	—	Pro	7
Lam CM^26^	China	1991–1997	159	252	63.1 (57.1,69.1)	31.5 ± 4.6	32.6 ± 4.9	—	32	—	Rep	8
Crane JM^5^	Canada	1988–1995	205	308	66.6 (61.3,71.8)	30.0	—	—	—	234	Rep	7
Dawson WB^35^	Canada	1991–1993	21	40	52.5 (37.0,68.0)	29.0 ± 5.0	—	—	—	—	Rep	6
Gorodeski IG^27^	Israel	1973–1983	99	155	63.9 (56.3,71.4)	—	—	—	18	—	Rep	7

AP: placenta on anterior wall; GA: gestational age; MP: multiperous; PCS: previous cesarean section; Pro: prospective cohort study; QS: quality score; Rep: retrospective cohort study.

^*^Values indicate the mean (range), or mean ± standard deviation.

**Table 2:** Results of meta-regression for the prevalence of antepartum hemorrhage.

Covariate	coefficient	95% CI	*t*-value	*P*-value
Age (year)	−0.0267	−0.0642–0.0108	−1.47	0.155
GA (week)	−0.0377	−0.0963–0.0209	−1.35	0.193
Survey year	−0.0101	−0.0191–0.0009	−2.25	0.033
AP	−0.1271	−0.8381–0.5839	−0.39	0.704
PCS	0.1093	−0.3571–0.5757	0.49	0.630
MP	0.6584	0.0791–1.2377	2.42	0.029
QS	0.0211	−0.0567–0.0988	0.56	0.583

AP: placenta on anterior wall; GA: gestational age; MP: multiperous; PCS: previous cesarean section; QS: quality score.

**Table 3:** The correlation between the mean prevalence and potential sources.

Covariate	Age (year)	GA (week)	Survey year	AP	PCS	MP	QS
r	−0.294	−0.301	−0.400	0.281	0.216	0.534	0.108
*P*	0.154	0.1975	0.031	0.330	0.334	0.031	0.578

AP: placenta on anterior wall; GA: gestational age; MP: multiperous; PCS: previous cesarean section; QS: quality score.
